# High dispersal capacity and biogeographic breaks shape the genetic diversity of a globally distributed reef‐dwelling calcifier

**DOI:** 10.1002/ece3.6335

**Published:** 2020-05-14

**Authors:** Martina Prazeres, Raphaël Morard, T. Edward Roberts, Steve S. Doo, Jamaluddin Jompa, Christiane Schmidt, Marleen Stuhr, Willem Renema, Michal Kucera

**Affiliations:** ^1^ Marine Biodiversity Group Naturalis Biodiversity Center Leiden The Netherlands; ^2^ MARUM University of Bremen Bremen Germany; ^3^ Leibniz Centre for Tropical Marine Research Bremen Germany; ^4^ Department of Biology California State University Northridge CA USA; ^5^ Hasanuddin University Makassar Indonesia; ^6^ Interuniversity Institute for Marine Sciences (IUI) Eilat Israel; ^7^ Bar‐Ilan University (BIU) Ramat Gan Israel

**Keywords:** biogeography, coral reefs, cryptic speciation, large benthic foraminifera, Lessepsian migrants, phylogeography

## Abstract

Understanding the role of dispersal and adaptation in the evolutionary history of marine species is essential for predicting their response to changing conditions. We analyzed patterns of genetic differentiation in the key tropical calcifying species of large benthic foraminifera *Amphistegina lobifera* to reveal the evolutionary processes responsible for its biogeographic distribution. We collected specimens from 16 sites encompassing the entire range of the species and analyzed hypervariable fragments of the 18S SSU rDNA marker. We identified six hierarchically organized genotypes with mutually exclusive distribution organized along a longitudinal gradient. The distribution is consistent with diversification occurring in the Indo‐West Pacific (IWP) followed by dispersal toward the periphery. This pattern can be explained by: (a) high dispersal capacity of the species, (b) habitat heterogeneity driving more recent differentiation in the IWP, and (c) ecological‐scale processes such as niche incumbency reinforcing patterns of genotype mutual exclusion. The dispersal potential of this species drives the ongoing range expansion into the Mediterranean Sea, indicating that *A. lobifera* is able to expand its distribution by tracking increases in temperature. The genetic structure reveals recent diversification and high rate of extinction in the evolutionary history of the clade suggesting a high turnover rate of the diversity at the cryptic level. This diversification dynamic combined with high dispersal potential, allowed the species to maintain a widespread distribution over periods of geological and climatic upheaval. These characteristics are likely to allow the species to modify its geographic range in response to ongoing global warming without requiring genetic differentiation.

## Graphical abstract

Genetic differentiation and distribution of populations can provide clues about processes shaping species evolutionary histories. We examined the genetic signature of globally distributed species of large benthic foraminifera in order to identify mechanisms acting at geological and ecological time scales. We found that while dispersal capacity allows populations to extend their range, geographic distribution was strongly shaped by biogeographic breaks and niche incumbency.

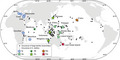

## 1 INTRODUCTION

Understanding the mechanisms that regulate the spatial distribution of species is fundamental to predict how individual taxa and ecosystems will respond to environmental changes (Evans, McKenna, Simpson, Tournois, & Genner, 2016). The current biogeography of species is the result of their evolutionary history, shaped by a combination of ecological niche preferences, biological interactions, and dispersal potential (Hellberg, 2009). These processes operate within the context of major tectonic changes occurring over geological time scales (Cowman & Bellwood, 2013; Keith, Baird, Hughes, Madin, & Connolly, 2013; Renema et al., 2008) making the modern‐day distribution of a species an integrated product of processes operating at different temporal and spatial scales. Ultimately, these processes regulate how and where species arise and how they respond to environment changes.

In species with limited dispersal, genetic differentiation may proceed by fragmentation of their habitat and local adaptation (Sanford & Kelly, 2011). Where dispersal is not limiting, genetic variation is a fundamental element of speciation (Pauls, Nowak, Balint, & Pfenninger, 2013). Genetic differentiation can arise due to adaptation to the local environment and encourage the emergence of ecologically enforced barriers to gene flow such as niche incumbency (Glor & Warren, 2011). Therefore, the assessment of intraspecific genetic diversity across broad spatial scales can provide valuable insights into the ecological and geological processes that create and maintain the genetic structure of populations.

Large benthic foraminifera (LBF) are crucial components of shallow marine ecosystems in tropical and subtropical environments worldwide (Langer & Hottinger, 2000). These prolific calcifiers are responsible for the production of a substantial portion of biogenic carbonate (up to 5%) on shallow marine shelves where accumulations of their sand‐grain‐sized shells contribute substantially to reef accretion and substrate stability (Langer, 2008). LBF harbor algal endosymbionts (reviewed in Prazeres & Renema, 2019) and show a strong species diversity maximum in shallow tropical warm environments (Förderer, Rodder, & Langer, 2018), and many species have wide geographic ranges, indicating high dispersal potential (Guastella et al., 2019; Langer & Hottinger, 2000). LBF utilize a wide range of dispersal mechanisms such as the passive transport of free‐swimming gametes into the water column during reproduction and of adult specimens, but also the formation of propagules (Alve & Goldstein, 2003). The formation of propagules provides an efficient mechanism for dispersal and might explain the wide distribution range in many benthic species (Alve & Goldstein, 2010).

The geographic range of LBF has been highly dynamic over geological time scales. Most notably, LBF fauna expanded their geographic ranges during past warm periods in the geologic past such as during the greenhouse warmth of the Eocene (50–33.9 Ma) as they tracked the movement of subtropical belts into higher latitudes (Adams, Lee, & Rosen, 1990; Hallock, 2000). These expansion episodes coincided with periods of radiation and diversification within LBF genera (Renema, 2015), including the acquisition of different algal symbionts (Prazeres & Renema, 2019), suggesting that changing environmental conditions together with the availability of new habitat could trigger diversification in this group (Hallock, Silva, & Boersma, 1991; Richardson, 2001). Observations (Caruso & Cosentino, 2014; Guastella et al., 2019) and model‐based projections of poleward range shifts suggest that many LBF species will benefit from current ocean warming (Weinmann, Rodder, Lotters, & Langer, 2013a, 2013b) as subtropical and temperate marine ecosystems become “tropicalised” (Verges et al., 2014). However, how poleward migration of LBF will affect local community assemblages and biogenic carbonate production is not known, as species are shifting their ranges beyond the normal glacial–interglacial oscillation range.

**FIGURE 1:**
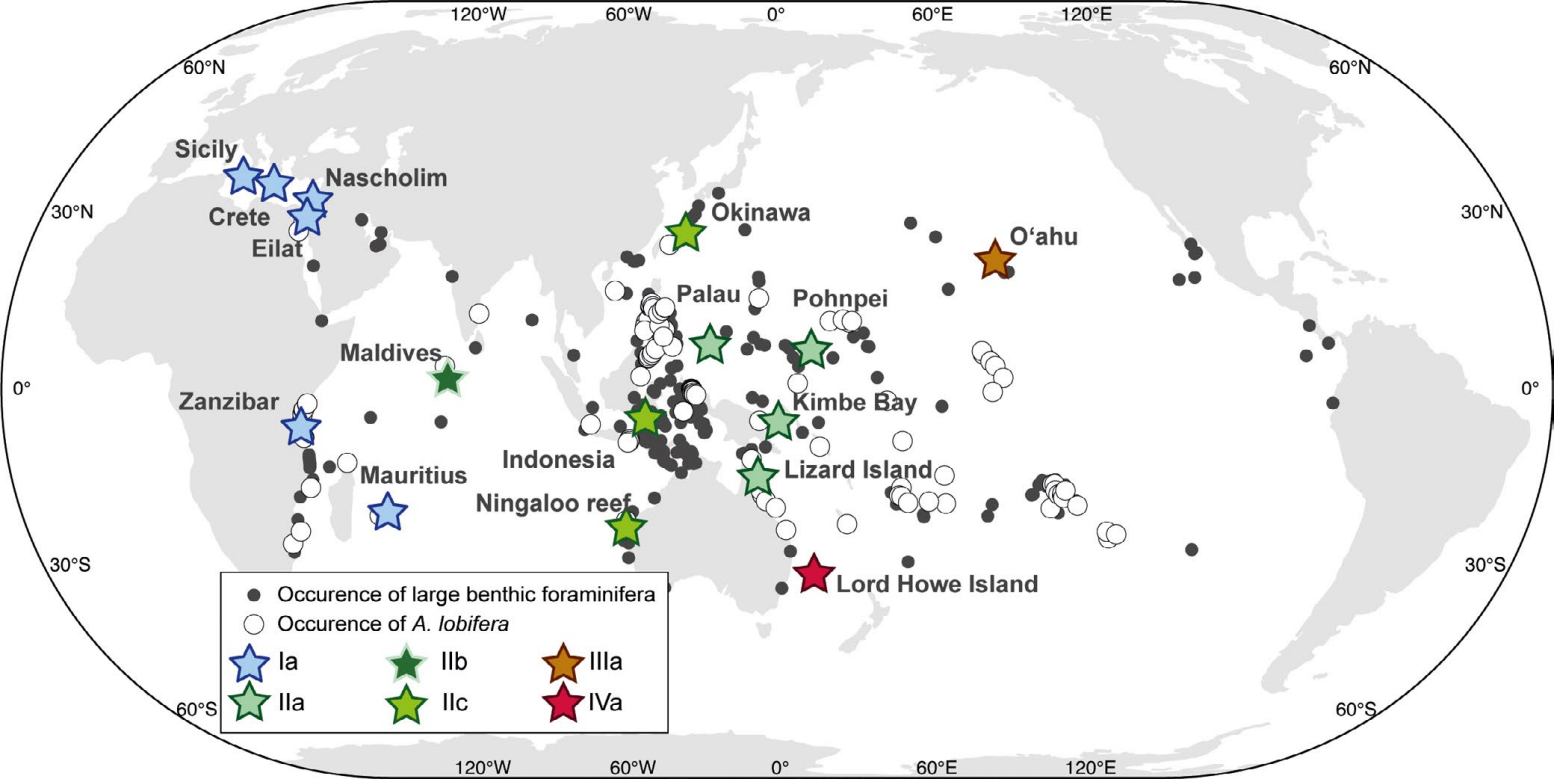
Global distribution of *Amphistegina lobifera*. The gray black dots indicate where assemblages of benthic foraminifera have been documented in the Indo‐Pacific region, Red Sea, and Mediterranean Sea (data from Förderer et al., 2018), and the open dots indicate where *A. lobifera* have been found (including the unaccepted species *A. madagascariensis,* which we consider here to be a subjective junior synonym of *A. lobifera*). Stars indicate the 16 collection sites where we obtained sequences of *A. lobifera* and colors show the occurrence of the genotypes of *A. lobifera* defined in Figure 2. Name of the sampling location is indicated next to the stars

In this context, it is crucial to understand the processes that generate genetic diversity in LBF and constrain the evolutionary legacy of their species. The information on spatial distribution of genetic diversity and the phylogenetic tree topology holds the key to understand the speciation process that shapes the distribution of these ubiquitous warm‐water protists. To investigate the current genetic structure of LBF, we selected the globally distributed symbiont‐bearing LBF species *Amphistegina lobifera*. This species is one of the most widespread extant LBF species, making it an ideal model to study the origin of genetic diversity in symbiont‐bearing calcifying organisms. *Amphistegina lobifera* is abundant throughout the central and western Pacific Ocean, Indian Ocean, and Red Sea (Förderer et al., 2018), and continues to successfully expand throughout the Mediterranean following the opening of the Suez Canal in 1869 (Guastella et al., 2019; Langer, Weinmann, Lotters, & Rodder, 2012). In this study, we assessed the genetic diversity of *A. lobifera* through the analysis of the 18S SSU rDNA, which is an established marker in foraminifera (Pawlowski & Holzmann, 2014; Weiner et al., 2016). We gathered samples within the known distribution range of *A. lobifera* (Figure 1), allowing us to study the pattern of genotype distribution across distinct environments and large geographic distances.

## 2 MATERIALS AND METHODS

### 2.1 Sample collection and preservation

In order to assess the genetic diversity and structure within *A. lobifera*, we collected living specimens in 13 reef localities distributed across the entire geographic range of the species (Figure 1). At each locality, pieces of reef rubble containing specimens of *A. lobifera* were collected from shallow habitats (0.2–6 m water depth) either through SCUBA diving or snorkeling using standard collection methods (Prazeres, Uthicke, & Pandolfi, 2016). Briefly, reef rubble pieces were placed in plastic bags, brought to the surface, and scrubbed using a brush. Resulting sediment was transferred to Petri dishes, where *A. lobifera* specimens could be identified. Specimens with uniform brown coloration and reticulopodial activity were selected and isolated in a micropaleontological slide or 1.5‐ml tube before being air‐ or oven‐dried overnight. Dried specimens were individually placed into empty 1.5‐ml tubes or micropaleontological slides, and taken to the laboratory at Naturalis Biodiversity Center, in the Netherlands. A minimum of eight specimens per site were selected for DNA extraction and amplification. The selected specimens were cleaned with 96% molecular grade ethanol under a stereomicroscope, and individual photographs were taken utilizing a Zeiss SteREO Discovery V12 stacking microscope. Individuals were subsequently placed in individual tubes containing 96% molecular grade ethanol for additional washing and removal of any contamination on the shell. To provide an out‐group to constrain the phylogeny within *A. lobifera*, specimens of the related species *A. lessonii* d’Orbigny were collected from the north shore of Mo'orea, French Polynesia (17°28.55′ S, 149°49.33′ W) with the same protocol as described above, and specimens where identified following Renema (2018).

### 2.2 DNA extraction, amplification, cloning, and sequencing

Total DNA of each individual foraminifera was extracted with the QIAamp^®^ DNA Micro Kit (Qiagen, Germany). Following the cleaning process, individual specimens were placed in 1.5‐ml tubes containing 200 µl of lysis buffer with added Proteinase K. DNA extractions of each specimen were then conducted according to manufacturer's instructions, and DNA concentration was quantified using the DropSense96 (Trinean, Belgium). After DNA extraction, between three and eight individual specimens per site generated enough DNA for downstream analysis. Total DNA concentration was standardized to 1 ng/µl of DNA across all samples. For amplification, we used a polymerase chain reaction (PCR) using the PHUSION^®^ Hot‐start II polymerase (Thermo Fisher Scientific, USA). We selected specific primers that targeted hypervariable regions in the 18S SSU rDNA. In all cases, DNA templates were amplified utilizing a seminested PCR approach, as the extracted DNA is likely to be dominated by genetic material from the symbionts, and the rDNA template within a single foraminiferal cell is low (Weiner et al., 2016). For amplification of the SSU rDNA, we used the primer sets: S14f3 (5′‐ACGCAMGTGTGAAACTTG‐3′) ‐ 1528R (5′‐TGATCCTTCTGCAGGTTCACCTAC‐3′) (Amaral‐Zettler, McCliment, Ducklow, & Huse, 2009; Pawlowski et al., 2002) and S14f1(5′‐AAGGGCACCACAAGAACGC‐3′) ‐ 1528R (de Vargas, Zaninetti, Hilbrecht, & Pawlowski, 1997), which amplifies a ~ 700 bp long region at the end of the SSU in *A. lobifera* (Schmidt, Morard, Prazeres, Barak, & Kucera, 2016). This fragment is traditionally used for barcoding benthic foraminifera (Pawlowski & Holzmann, 2014). For amplification, we used a mix containing 2 µl of DNA extract with 0.5 µM of each primer, 3% DMSO, 1x Phusion Green buffer, 0.5 µM dNTP, 1.25 µM MgCl_2_, and 0.2 units of polymerase in a final volume of 20 µl. The PCR profile for amplification using the [S14f3‐1528R] and [S14f1‐1528R] pair was as follows: initial denaturation at 98°C for 3 min, 30 cycles of 30 s of denaturation at 98°C, annealing for 30 s at 62°C for the [S14f3‐1528R] primer set, and 67°C for the [S14f1‐1528R] primer couple, and extension for 15 s at 72°C, followed by a final extension of 5 min at 72°C. PCR products were checked visually on 1% agarose gels under UV light and subsequently purified using the PureLink™ PCR Purification Kit (Invitrogen, USA) following manufacturer's instructions.

The purified PCR product was cloned using the TOPO^®^ TA Cloning Kit (Invitrogen, USA). Amplicons were ligated to a pCR 2.1‐TOPO^®^ vector, transformed into One Shot™ TOP10 chemically competent *Escherichia coli* cells, and grown overnight on LB‐agar plates containing ampicillin (50 mg/ml). Eight to 16 clones per specimen were selected and placed in 0.5‐ml tubes containing 18.2 Ω MilliQ water. Bacterial cells were lysed through one freezing–thawing cycle, and a final PCR was performed using the primer set [S14f1‐1528R]. Positive PCR products were sequenced in both directions using an ABI 3730xl DNA Analyzer (Thermo Fisher Scientific, USA) at BaseClear (Leiden, Netherlands). The obtained chromatograms were manually checked, complementary fragments of the same sequence were de novo assembled, primer sequences removed from both ends, and consensus sequences were deposited on NCBI under the accession number TBA.

To complete our dataset, we retrieved 25 sequences of *A. lobifera* from NCBI GenBank (Schmidt et al., 2016) together with their metadata. These sequences were generated from nine specimens collected at three additional locations: two in the Mediterranean Sea (Crete, Greece and Nahsholim, Israel) and one in the Red sea (Eilat, Israel), in addition to samples previously collected from eastern Australia (Lizard Island, on the Great Barrier Reef). As a result, our dataset included sequenced specimens from 16 collection sites. Sequences metadata included in the study and accession numbers are provided as Appendix S1.

### 2.3 Genetic variability and phylogeny of *A. lobifera* genotypes

To evaluate the extent of genetic variability within *A. lobifera*, we applied the molecular taxonomic system described by Morard et al. (2016). Briefly, the system is organized in three hierarchical levels below the morphospecies classification: basegroups, genotypes, and lineages. Basegroups represent the lowest level of classification, followed by genotypes, and lineage, which is the highest levels of molecular taxonomy. Basegroups consist of basetypes, which are unique sequences within each single specimen. Where multiple genes are available for single individuals, each sequence pattern for each locus is a basetype (Morard et al., 2016). We have selected basetypes that occur at least twice in our dataset. Basetype sequences were automatically aligned with MAFFT v7 (Katoh & Standley, 2013). Genotypes and lineages were delineated using a combination of two automated delimitation methods, the Automated Barcode Gap Discovery method (ABGD) (Puillandre, Lambert, Brouillet, & Achaz, 2012) and the Poisson Tree Process (PTP) (Zhang, Kapli, Pavlidis, & Stamatakis, 2013). Genotypes represent the level of biological species, while lineages represent a major disruption in the genetic variability within a given morphospecies organized in monophyletic clusters consisting of one or several genotypes (Morard et al., 2016). We calculated two phylogenetic inferences from two alignments utilizing basetypes, with and without sequences of *A. lessonii* that served as an out‐group. Each phylogenetic inference was carried out with 1,000 nonparametric bootstrapping pseudoreplicates based on a BioNJ starting tree using PhyML (Guindon, Dufayard, Lefort, Anisimova, Hordijk, & Gascuel, 2010), and the best substitution models were selected using the Smart Model Selection (Lefort, Longueville, & Gascuel, 2017) under Akaike information criterion. The model GTR + G + I was retained for both inferences. Resulting trees were submitted on the PTP server (http://species.h‐its.org/). Default options were selected for both trees, and in one of them, *A. lessonii* was indicated as an out‐group. We retained the delimitation returned by the maximum‐likelihood solution. The resulting tree was visualized with iTOL v4 (Letunic & Bork, 2019). The resulting molecular nomenclature was validated by calculating patristic distances on the unrooted tree using SeaView v4.7 (Gouy, Guindon, & Gascuel, 2010) and comparing the distance gaps that are expected to occur between the successive hierarchal levels (Lefebure, Douady, Gouy, & Gibert, 2006). Patristic distances were compared using the Kolmogorov–Smirnov and Mann–Whitney tests implemented in PAST v3.21c (Hammer, Harper, & Ryan, 2001), and the results are reported in Appendix S2. Where the partition returned by ABGD and/or PTP was invalidated by the patristic distance, the partition was merged into the closest neighboring unit following the patristic distance. Detailed description of molecular taxonomy and construction of phylogeny tree can be found in the Appendix S1. The phylogenetic tree and associated molecular taxonomy are shown in Figure 1b.

### 2.4 Rate of diversification

We investigated the rate of diversification of basetypes in order to constrain the patterns of diversification of *A. lobifera*. We transformed the unrooted maximum‐likelihood tree into an ultrametric tree using a relaxed clock model to show the relative time of branching of the basetypes using the packages *ape* (Paradis, Claude, & Strimmer, 2004), *phytools* (Revell, 2012), and *ggtree* (Yu, Smith, Zhu, Guan, & Lam, 2017) in R (R Core Team, 2018). We plotted the lineages through time (LTT) plot of the entire tree and the lineages I and II separately together with the rate of diversification Pybus *γ* calculated for each clade (Figure 3a; (Pybus & Harvey, 2000) using the *nLTT* package (Janzen, Höhna, & Etienne, 2015). It was not possible to assess the rate of diversification in lineages III and IV because these lineages had only one and two basetypes, respectively. We then calculated 100 random trees under a pure‐birth model using the parameters of the entire *A. lobifera* clade and the lineages I and II separately. The pure‐birth model assumed only production of lineages through time and no extinction (Pybus, Rambaut, Holmes, & Harvey, 2002). The Pybus γ statistic assumes that under a pure‐birth process the distribution of γ follows a standard normal distribution. Therefore, deviations from a log‐linear increase can be used to reject a constant, pure‐birth model of diversification, and might be used to infer the rate of historical variation (Fordyce, 2010). We plotted the LTT of the entire *A. lobifera* clade and the lineages I and II separately to compare them with those generated under the pure‐birth model (Figure 3b). Finally, we calculated 1,000 random trees using the same properties that the lineages I and II calculate the Pybus γ of the simulated trees of both clades that were subsequently compared with a nonparametric Wilcoxon test (Figure 3c). The letter‐value (L‐V) plot displaying the Pybus γ (Figure 3c) was generated using the L‐V plot package (Hofmann, Wickham, & Kafadar, 2017).

### 2.5 Relationship between patristic and geographic distances

Median patristic distances within and between genotypes were calculated and compared by applying a pairwise *t* test using the Bonferroni p‐value adjustment method using the package *stats*, which is implemented within the software R (R Core Team, 2018), except for the lineage III that consists of a single basetype. The low variability in patristic distance within lineage I, and high patristic distance between lineages II and III, which are geographically close, led us to test the influence of speciation by distances (Mayr, 1942). We calculated the geographic distances between all pairs of localities (*n* = 78) using the Haversine formula in MATLAB v. R2017b. However, we calculated distances between O’ahu and Sicily, and O’ahu and Crete manually using the tracking tool in Google Earth going through the Equator. The Haversine formula calculates the shortest distance over the Earth's surface between two geographic points, and so it calculated these distances over the poles, which is not a relevant ecological representation of the distances between the sites in the Mediterranean and the Pacific Ocean. We estimated the relationship between the patristic genetic distance and geographic distance between all pairs of genotypes averaged per locality using a linear regression model (Appendix S3). Pairs where genetic distance was significantly distinct from the linear regression value were identified by applying a confidence interval to the regression, consisting of the mean range of patristic distances calculated within each pair recorded. Values within the confidence interval were then considered to be within the expected range for a speciation by distance model. Pairs where genetic distance was lower than expected considering the geographic distance are referred to here as genetically underdispersed. Conversely, pairs where genetic distance was higher than expected from geographic distance are referred to as genetically overdispersed. The correlation matrix between patristic and geographic distances was plotted in R (R Core Team, 2018) using the package *ggplot2* (Wickham, 2009).

## 3 RESULTS

### 3.1 Genetic diversity

In total, we analyzed 453 SSU sequences, belonging to 77 specimens (Appendix S1). Of the 453 sequences, 210 could be used to define a total of 109 basetypes, constituting 29 basegroups. In the phylogeny rooted on the sister species *A. lessoni*, all sequences of *A. lobifera* clustered together with maximum branch support (Figure S1). Both unrooted and rooted phylogeny returned a basic topology with the *A. lobifera* sequences organized in four clusters. Although they obtained only moderate bootstrap support between 50% and 98.5% in the unrooted and rooted phylogenies, respectively, these four clades were identified as putative species by the initial partitioning of ABGD and the PTP analyses performed on the rooted tree. The four clusters were statistically validated using patristic distances (Appendix S2) and were therefore considered as lineages (I, II, III, and IV) in our nomenclature. However, the recursive partitions of ABGD and the PTP analysis carried out on the unrooted tree returned 36 and 21 inconsistent partitioning that oversplit basegroups. The partition that split sequences belonging to the same basegroups was aggregated, resulting in seven partitions returned by ABGD and five partitions by the PTP analysis. A single genotype was identified within each of the lineages I, III, and IV, but up to four putative genotypes (IIa, IIb, IIc, and IId) were identified within lineage II. Three of the four partitions were statistically validated (IIa, IIb, and IIc), while the putative genotype IId, constituted by a single sequence, was not supported (Appendix S2) and was subsequently merged with the genotype IIc and considered as the basegroup IIc6. No differences were identified between the intra‐ and interbasegroup levels within each genotype, attesting to the genetic homogeneity of each genotype despite the high intragenotype variability (Figure 4). Finally, we assigned the remaining 243 sequences to the previously defined genotypes in order to maximize our coverage for the ecological inference and plotted the resulting biogeography in Figure 1.

### 3.2 Spatial distribution of genotypes

**FIGURE 2:**
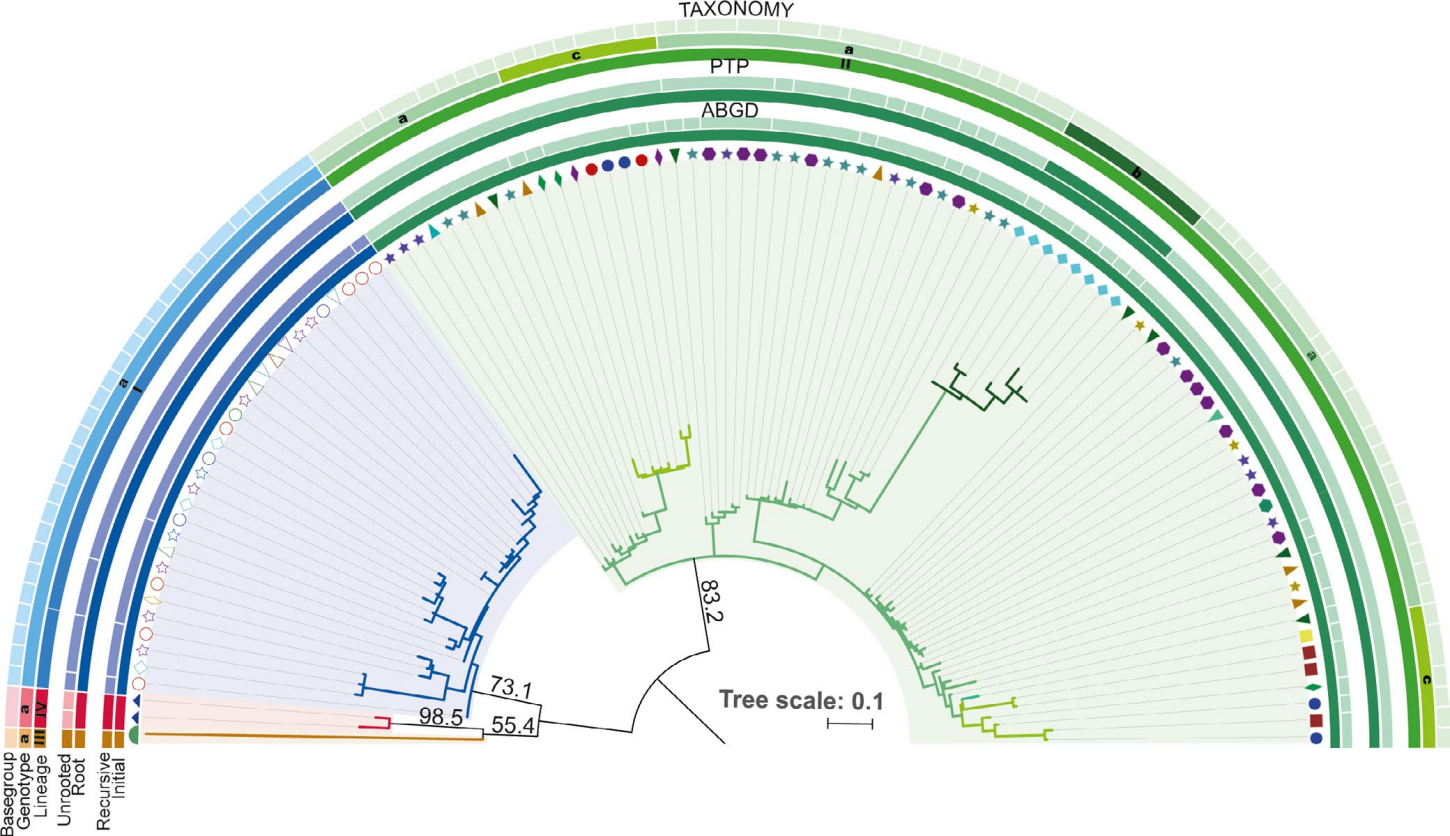
Molecular taxonomy of *A. lobifera*. Each branch represents a unique basetype, and the symbol next to the branch represents the individual basegroup. The four inner‐most set of rings represent the delineation proposed by the Automated Barcode Gap Discovery (ABGD) and the Poisson Tree Process (PTP) methods, the lighter colors representing when the delineation was invalidated because of over splitting of basegroups (see results). The three outer‐most rings represent the final nomenclature retained with the three hierarchal levels represented successively. Bootstrap support values are only indicated for the lineages, and the topology within the lineages is largely unsupported

No co‐occurrences of genotypes were observed in the dataset, suggesting that the genotypes are mutually exclusive (Figure 1). Lineage I consists of a single genotype spanning a geographic range of 7,500 km, across the western Indian Ocean, Red Sea, and the Mediterranean Sea. Lineage II comprises of three genotypes and ranges from the Maldives to the Pacific Island of Pohnpei, in the Federated States of Micronesia (Figure 2). A longitudinal gradient is apparent within lineage II. Genotype IIa occupies the eastern part of the biogeographic range and was observed at four sites (Pohnpei, Palau, Lizard Island, and Kimbe Bay). Genotype IIb was only observed in the Maldives, while genotype IIc occupied the central part of the lineage range and was found in Ningaloo reef, Indonesia, and Okinawa in Japan. Finally, lineages III and IV are restricted to the isolated islands of O’ahu in Hawai'i, and Lord Howe, respectively (Figure 1).

### 3.3 Rates of diversification

**FIGURE 3:**
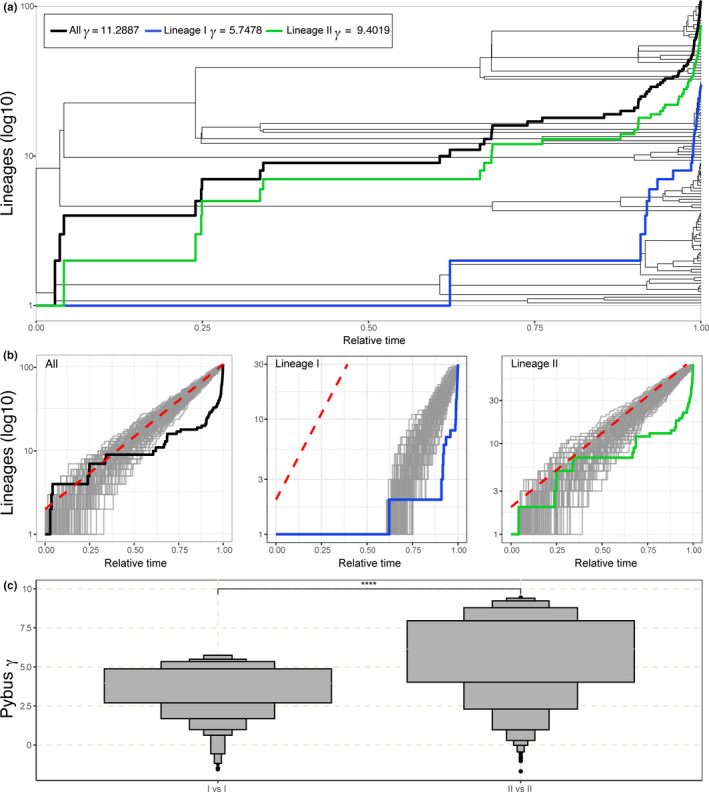
(a) Ultrametrized tree showing diversification of *A. lobifera* against relative time. Colored lines represent the lineage through time (LTT) plot for the entire clade, lineage I, and lineage II, combined with the indicated rate of diversification for each clade. (b) LTT plots of randomly generated trees under a pure‐birth model for all *A. lobifera,* lineage I, and lineage II. Colored solid lines show observed LTT and the deviation from the pure‐birth model. (c) Letter‐value (L‐V) plot displaying the Pybus γ calculated on random trees generated using the same properties as lineages I and II. The stars above the horizontal line on top of the L‐V plot represent the level of significance of the Wilcoxon test that compared the means of both distributions

Based on the LTT plot, lineage I displays shallower diversification than lineage II (Figure 3a). Pybus γ values calculated either on the entire tree, or for lineages I and II separately, are all above 0 meaning that the internal nodes of the clades are closer to the tips than from the deepest nodes of the tree, which is indicative of a strong divergence from the pure‐birth model (Pybus & Harvey, 2000). Lineage II showed a higher divergence from the pure‐birth model than lineage I, even though it consists of three genotypes. This is indicative of a high death rate (linage disappearance), which prevents retention of deep branching, and thus of ancient clades in the phylogeny. This impression is reinforced when comparing the LTT plots of each lineage with the pure‐birth model (Figure 3b). We observed an overall divergence of *A. lobifera* phylogeny from the pure‐birth model toward fewer lineages occurring between 0.3 and 0.6 of the relative age of the clade. A deviation from the pure‐birth model is also observed when the two lineages are analyzed separately, but the deviation from the pure‐birth model is weaker in lineage I. The Pybus γ values calculated on random trees generated with similar properties than lineages I and II show that both lineages underwent different diversification processes (Figure 3c, Wilcoxon test of Pybus γ; *p* = 3.40E‐95).

### 3.4 Speciation by distance

**FIGURE 4:**
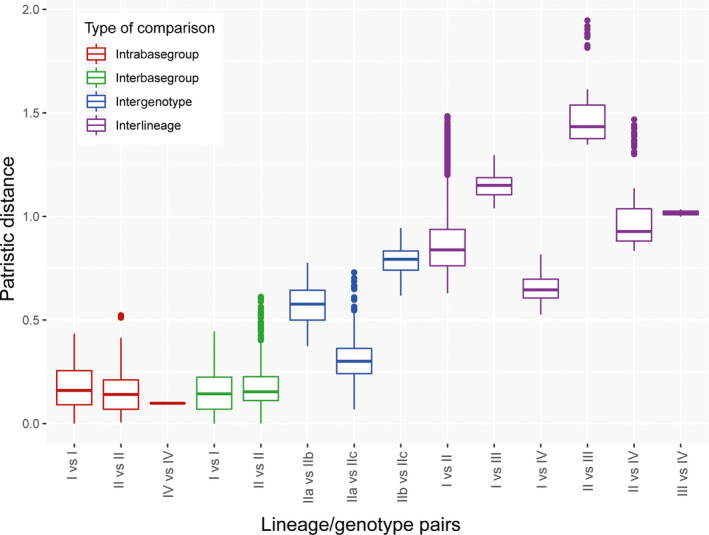
Median patristic distance within and between lineages/genotype. Boxes represent quartiles, whiskers represent smallest and largest values within 1.5 times interquartile range, and dots are outliers. Note that patristic distance within genotype IIIa cannot be calculated as it consists of a single basetype

**FIGURE 5:**
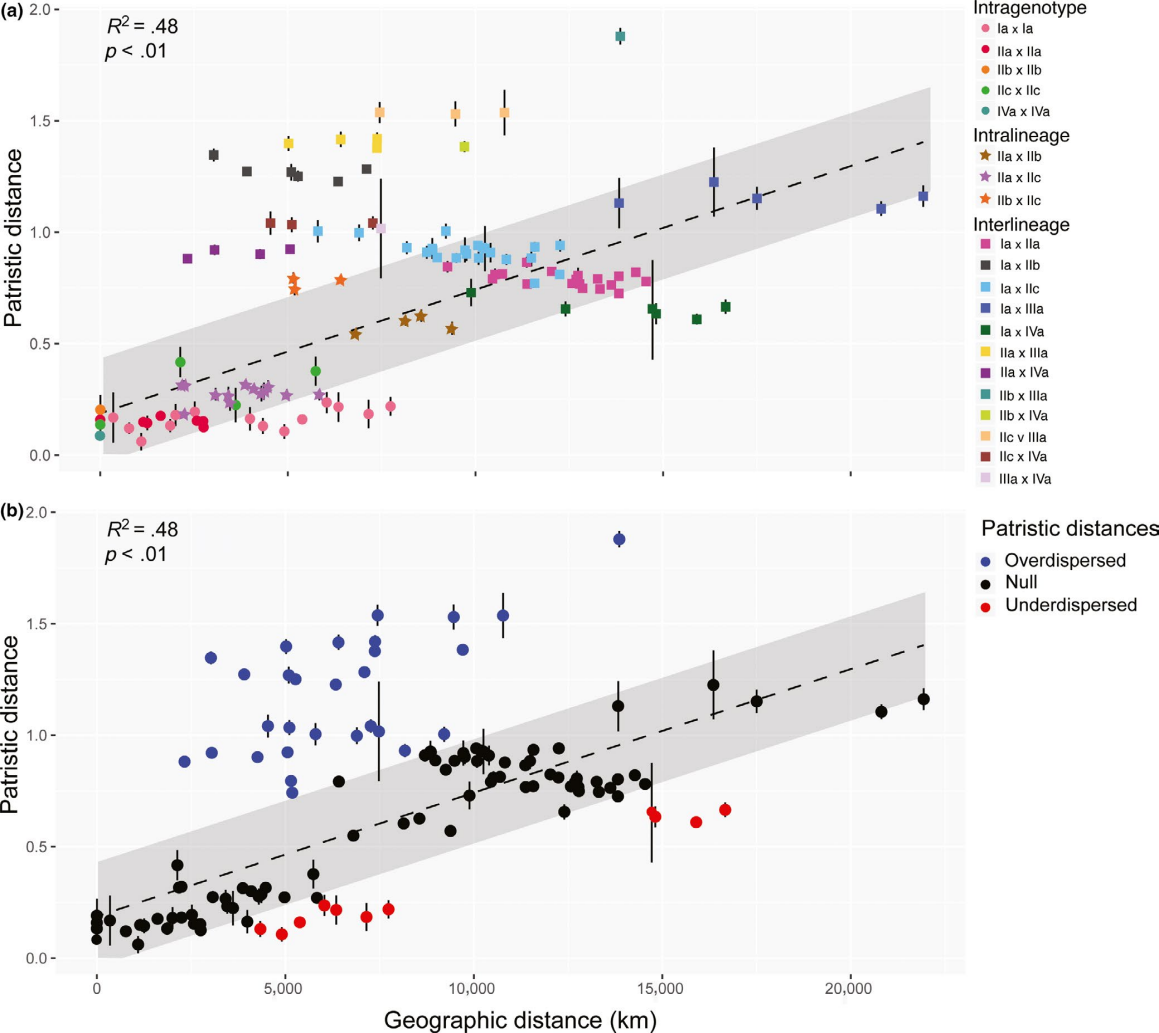
Linear correlation between patristic distance as a function of geographic distance within and between genotypes of *A. lobifera* (*R*‐squared: .48; *p* < .01). Points represent mean patristic distance between each of the 78 different site combinations. Bars indicate minimum and maximum values within each site pair. Diagonal line represents a linear regression, and the gray polygon reflects the confidence interval. (a) Site pairs are colored‐coded by genotypes represented within the pair. Circles, stars, and squares represent intragenotype, intralineage, and intergenotype comparison, respectively. (b) Blue and red circles represent site pairs that are statistically underdispersed and overdispersed, respectively. Pairs where the mean patristic distance lies within the confidence interval are represented by black dots

Genotypes IIb, IIIa, and IVa, which are restricted to oceanic islands, possess low genetic variability (Figures 2 and 4). In contrast, genotypes Ia, IIa, and IIc are widespread and displayed higher genetic variability (Figure 4). This observation led us to test for the hypothesis of speciation by distance (Figure 5). This analysis revealed an overall positive relationship between geographic and patristic distances (*R* ^2^ = .48, *p*‐value < .01; Figure 5), but many of the pairwise comparisons deviated significantly from the overall regression model. For example, patristic distance between Sicily and Mauritius indicates statistical underdispersion indicative of an efficient genetic mixing, while distances between Lord Howe and Lizard Islands are statistically overdispersed (Figure 5) indicative of the absence of gene flow and thus divergence between these two lineages even with geographic proximity. Overall, pairs that were statistically overdispersed consistently featured genotypes IIIa and IVa, which are found in the isolated islands of O'ahu and Lord Howe. Conversely, statistically underdispersed pairs all feature genotype Ia, while genotypes in lineage II largely follow the expected trend of the regression model (Figure 5).

## 4 DISCUSSION

The existence of multiple distinct genotypes within the global range of *A. lobifera* demonstrates that the species underwent repeated episodes of diversification, which remained morphologically cryptic. Lineage I has an extensive geographic range and demonstrates the high dispersal capacity of *A. lobifera*, while lineages III and IV are limited to isolated oceanic islands. The most remarkable pattern of the diversification is the large degree of mutual exclusion among genotypes. A similar pattern has also been observed in planktonic foraminifera (Aurahs, Grimm, Hemleben, Hemleben, & Kucera, 2009; Weiner et al., 2014), and it is consistent with niche partitioning. The highly heterogeneous microhabitats within the Indo‐Australian Archipelago (IAA) (Lohman et al., 2011) likely contribute to the observed patterns of genetic divergence within the geographically constrained lineage II and suggest that the generation of diversity in *A. lobifera* is stimulated within this region. These findings support the hypothesis that the IAA acts as an evolutionary incubator of diversity (e.g., Bowen et al., 2016).

### 4.1 Dispersal capacity of A. lobifera

Our results show that *A. lobifera* is capable of expanding its range over large geographic distances. However, it does not appear to be able to maintain genetic homogeneity over its entire geographic range, as evidenced by the longitudinal genetic differentiation (Figure 1). The extensive observed dispersal capacity, best documented by lineage I, should be sufficient to allow the individual lineages and genotypes of *A. lobifera* to track the periodic expansion of the subtropical belt during Quaternary ice age cycles without requiring speciation. This hypothesis is consistent with the recent colonization of the Mediterranean Sea after the opening of the Suez Canal (Triantaphyllou, Dimiza, Koukousioura, & Hallock, 2012) from the Indian Ocean by lineage I without any sign of genetic divergence. It appears that lineage I was able to reinvade the Red Sea when the habitat became available after the last glacial salinity crisis (Biton, Gildor, & Peltier, 2008). Subsequently, *A. lobifera* was able to extend its range into the Mediterranean once the geographic barrier was removed following the opening of the Suez Canal. The two isolated marginal seas were therefore colonized without the establishment of genetically distinct local population. In the western Indian Ocean, the presence of the South Equatorial current moving westwards and the northward East African current likely facilitates the homogenization of populations along the African coast and Mauritius. In combination, these processes likely created and maintain the patterns of distribution observed in lineage I.

### 4.2 Fragmentation of habitat and generation of genetic diversity

In contrast to the homogeneous lineage I in the western Indian Ocean, and Red and Mediterranean Seas, lineage II has diversified across the central Indian and Pacific Oceans.

LTT plots show that lineage II diverged significantly from the pure‐birth model (Figure 3a,b). Further, the calculated Pybus γ values show that lineage II has a higher divergence from the model than lineage I (Figure 3c), suggesting that there is higher death rate concurrent with the high diversification in lineage II. This result indicates that the processes which generate genetic diversification are also inclined to support higher rates of extinction. The genetic diversification in the Indo‐Pacific can be a result of the increase of coral associated environment in that region (Renema et al., 2008; Wilson & Rosen, 1998). The development of the IAA and the formation of extensive reef flat areas created heterogeneous shallow environments (Keith et al., 2013; Santodomingo, Renema, & Johnson, 2016), which facilitate ecological specialization and the emergence of genetic divergences within lineage II. This process of divergence was likely further reinforced by a suppression of gene flow, probably during sea level variations caused by the glacial cycles of the Pliocene and Pleistocene (Naish et al., 2009). This divergence has been subsequently maintained to the present day in the IAA, with the three genotypes within lineage II remaining distinct following the fragmentation of the ancestral population. As a result, genetic differences observed between genotypes IIa and IIc, which are separated by 2,500–6,000 km, thus below the extension of 7,500 km observed within the lineage I, cannot be explained by geographic separation alone.

Within genotype IIa, the North Equatorial Counter Current, Equatorial Counter Current, and East Australian Current (Wijeratne, Pattiaratchi, & Proctor, 2018) allow continuing gene flow between *A. lobifera* populations from reefs in Papua New Guinea, Palau, the northern Great Barrier Reef (Lizard Island), and Pohnpei. Similarly, the genotype IIc occurring in Ningaloo reef, Okinawa, and Indonesia is connected through the Leeuwin Current that allows genetic mixing of populations occurring in the Pacific and Indian Oceans (Wilson & Kirkendale, 2016). Additionally, the Mindanao Current and Kuroshio Currents connect populations from reefs in Okinawa and Makassar (Indonesia), and create barriers between genotypes IIa and IIc. Importantly, geographic patterns of diversity observed in our study follow the same genetic diversity distribution patterns found in other marine populations, (e.g., Coleman et al., 2016; Otwoma & Kochzius, 2016; Williams, Jara, Gomez, & Knowlton, 2002), where there is a clear genetic break between the populations from the Indian and Pacific Oceans.

We found genotype IIb only in the Maldives (Figures 1 and 2) and cannot assess the extent of its biogeographic range. However, our phylogenetic results suggest that genotype IIb originated within the IAA similarly to the genotypes IIa and IIc. This pattern is consistent with biogeographic boundaries of LBF communities within the Indian Ocean, where communities in the Maldives and Indo‐West Pacific (IWP) are more similar than those found in the west Indian Ocean (Langer & Hottinger, 2000; Parker & Gischler, 2011). The differentiation into genotype IIb is consistent with limited water movement between Pacific and Indian Oceans during glacial low sea levels (Gaither & Rocha, 2013; Horne, 2014), thus restricting gene flow after initial colonization of the Indian Ocean from the IAA.

### 4.3 Deep divergences and historical biogeography

We identified two endemic lineages (III in O’ahu and IV in Lord Howe Island), which showed significant divergence from each other in the central Pacific region (Figures 1 and 2). This pattern of genotype isolation in Lord Howe and Hawai'i Islands has also been observed in corals (Ayre & Hughes, 2004; Baums, Boulay, Polato, & Hellberg, 2012). Patristic distance analysis demonstrated that geographic distance cannot explain differences between genotypes IIa and IIIa/IVa, which are geographically close. The genetic differentiation and emergence of lineages III and IV are instead consistent with deep historical divergence rather than a mere speciation by distance. A likely scenario is that these isolated islands were colonized during the Pliocene when conditions were warm and subtropical belts extended to high latitudes, consistent with dispersal patterns and speciation of other species of LBF (Faichney et al., 2011; Renema, 2015). Due to its high dispersal capacity, *A. lobifera* was able to migrate from the IAA to mid‐latitudes and distantly isolated islands in the Pacific, before land masses reduced ocean circulation in the IAA (Springer & Williams, 1990). Following the initial colonizers, reduced gene flow between these isolated islands and the IAA or ecological specialization in these habitats allowed the initial invaders to drift genetically. Both locations coincide with regions that experienced little change in temperature (and presumably other key environmental parameters) during Quaternary cycles as indicated by glacial temperature reconstructions for the last glacial maximum (Annan & Hargreaves, 2013), allowing the species to remain in their habitat without further diversification, which is also consistent with the low genetic variability found within both the O’ahu and Lord Howe Island populations. With time, these isolated populations have potentially adapted to their local habitats (e.g., Prazeres, Roberts, & Pandolfi, 2017), allowing the existing population an advantage over new immigrants, and further reinforcing the genetic barrier by incumbency (Barton & Charlesworth, 1984; De Meester, Vanoverbeke, Kilsdonk, & Urban, 2016). Under this scenario, the south‐eastern portion of the distribution range of *A. lobifera* that has not been assessed may shelter similar relict lineages from the initial expansion of the species.

### 4.4 Ongoing ocean warming and range expansion in *A. lobifera*

Rapid climate change is predicted to affect the distribution of many marine species by forcing them into either contracting or expanding their distributions (Hiddink, Lasram, Cantrill, & Davies, 2012; Pearson & Dawson, 2003). The successful colonization of many alien species into the Mediterranean Sea following the opening the Suez canal has been suggested to be a “best‐case” assessment of the effects of climate change on marine biodiversity (Hiddink et al., 2012). This is because the Mediterranean Sea is experiencing ongoing temperature rise, which facilitates the migration of tropical species from the Red Sea, thus representing a model system for understanding global patterns of species distribution in other larger marine ecosystems (Lejeusne, Chevaldonne, Pergent‐Martini, Boudouresque, & Perez, 2010). Within the tropical benthic foraminifera species, *A. lobifera* is a successful Lessepsian migrant, colonizing the eastern Mediterranean Sea (El Kateb, Stalder, Stainbank, Fentimen, & Spezzaferri, 2018; Triantaphyllou et al., 2012). There is a suggestion that *A. lobifera* reached the eastern Mediterranean Sea in the Holocene (ca. 6 ka), much earlier than the opening of the Suez Canal in 1869 through a different natural water way connecting Indo‐Pacific to the Eastern Mediterranean (Meric et al., 2016). However, our results indicate that the current populations in the Mediterranean Sea and along the coast of east Africa are genetically mixed (Figures 1 and 2), precluding the possibility that the Mediterranean population is a relic from a previous isolation.

The expansion of *A. lobifera* into the Mediterranean Sea is suggested to be limited by their observed thermal distributional limit of ~13–14°C (Guastella et al., 2019; Larsen, 1976). Therefore, it is likely that instead of requiring genetic differentiation to facilitate invasion, *A. lobifera* is expanding in pace with its thermal niche (Caruso & Cosentino, 2014). Additionally, the capacity to tolerate high temperatures (32–33°C) can be a crucial conserved trait carried by the populations from the Red Sea into the Mediterranean Sea (Schmidt et al., 2016; Titelboim et al., 2019). Such pre‐adaptive traits to higher temperatures confer *A. lobifera* a clear adaptive advantage in shallow and episodically high temperature environments in the Mediterranean Sea under continuing warming scenarios (Schmidt et al., 2016). In this case, ongoing ocean warming is likely to induce further range expansion at the peripheral populations.

## CONFLICT OF INTEREST

The authors declare no conflicting interests.

## AUTHOR CONTRIBUTIONS

**Martina Prazeres:** Conceptualization (lead); Data curation (lead); Formal analysis (lead); Funding acquisition (lead); Investigation (lead); Methodology (lead); Project administration (lead); Writing‐original draft (lead); Writing‐review & editing (lead). **Raphaël Morard:** Conceptualization (supporting); Data curation (supporting); Formal analysis (equal); Methodology (equal); Writing‐review & editing (supporting). **Thomas Edward Roberts:** Conceptualization (supporting); Data curation (supporting); Formal analysis (supporting); Writing‐review & editing (supporting). **Steve S. Doo:** Data curation (supporting); Writing‐review & editing (supporting). **Jamaluddin Jompa:** Resources (supporting); Writing‐review & editing (supporting). **Christiane Schmidt:** Data curation (supporting); Writing‐review & editing (supporting). **Marleen Stuhr:** Data curation (supporting); Writing‐review & editing (supporting). **Willem Renema:** Conceptualization (supporting); Data curation (supporting); Writing‐review & editing (supporting). **Michal Kucera:** Conceptualization (supporting); Supervision (lead); Validation (supporting); Writing‐review & editing (supporting).

## Supporting information

Figure S1

Appendix S1

Appendix S2

Appendix S3

## Data Availability

All relevant data are available within the manuscript Supporting Information files, and sequences are deposited on the GenBank public repository under the accession numbers MN831967 to MN832422.
