# Supplementary figures and images for: High dispersal capacity and biogeographic breaks shape the genetic diversity of a globally distributed reef‐dwelling calcifier
Source: Ecol Evol. 2020 May 14;10(12):5976–89. doi: 10.1002/ece3.6335 (PMC7319125; doi:10.1002/ece3.6335)

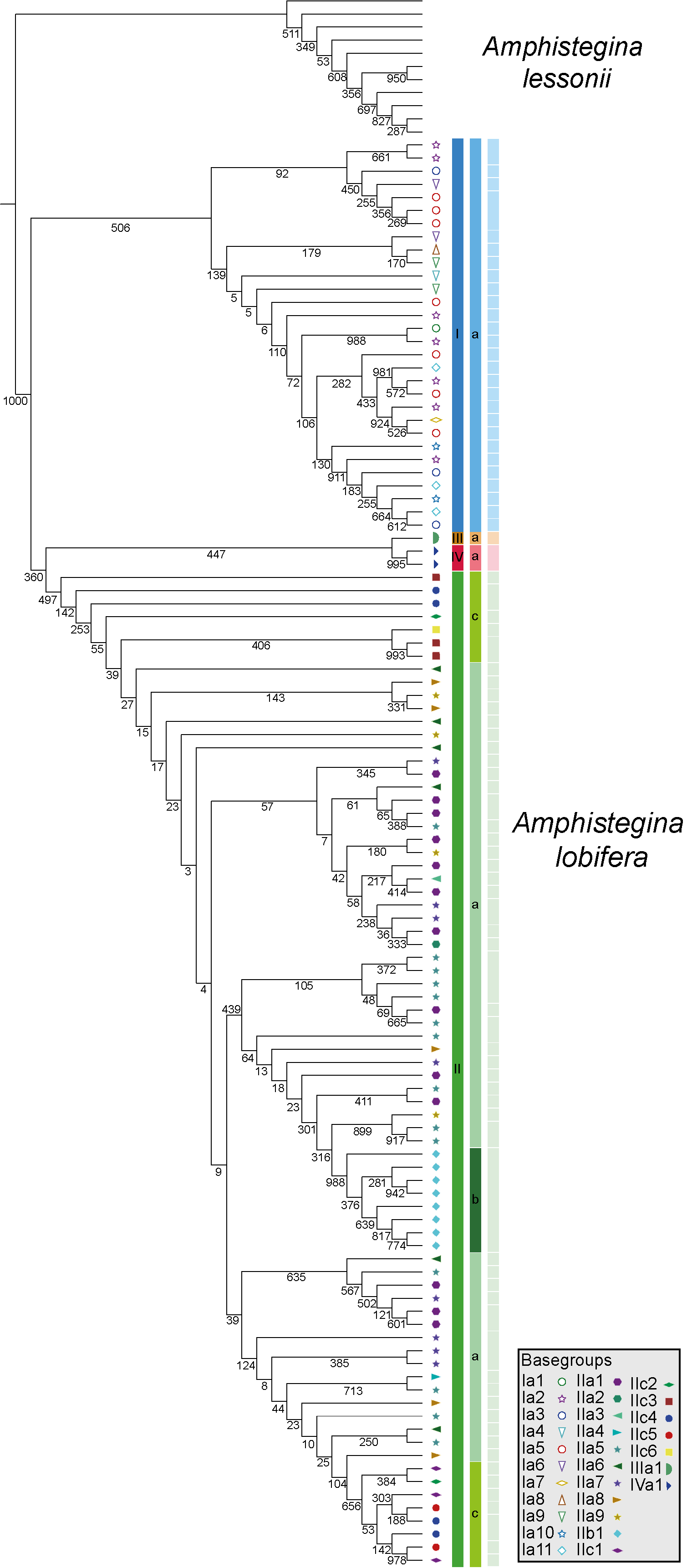

Supplement: Supplementary file 1 — Figure S1 [file ECE3-10-5976-s001.tif]
